# Supplementary material for: H pilin cyclisation and pilus biogenesis are promiscuous but electrostatic perturbations impair conjugation efficiency
Source: Nat Commun. 2026 Feb 18;17:2888. doi: 10.1038/s41467-026-69599-3 (PMC13031515; doi:10.1038/s41467-026-69599-3)
Supplement: Supplementary file 1 — Supplementary Information [file 41467_2026_69599_MOESM1_ESM.docx]

**a**

**b**

**c**

**Supplementary Figure 1.** Mapping of the ThrA mutant side chains on the surface of the assembled pilus. (a) D69A, (b) D69G and (c) D69N. Colour scheme as in Figure 4.

**a**
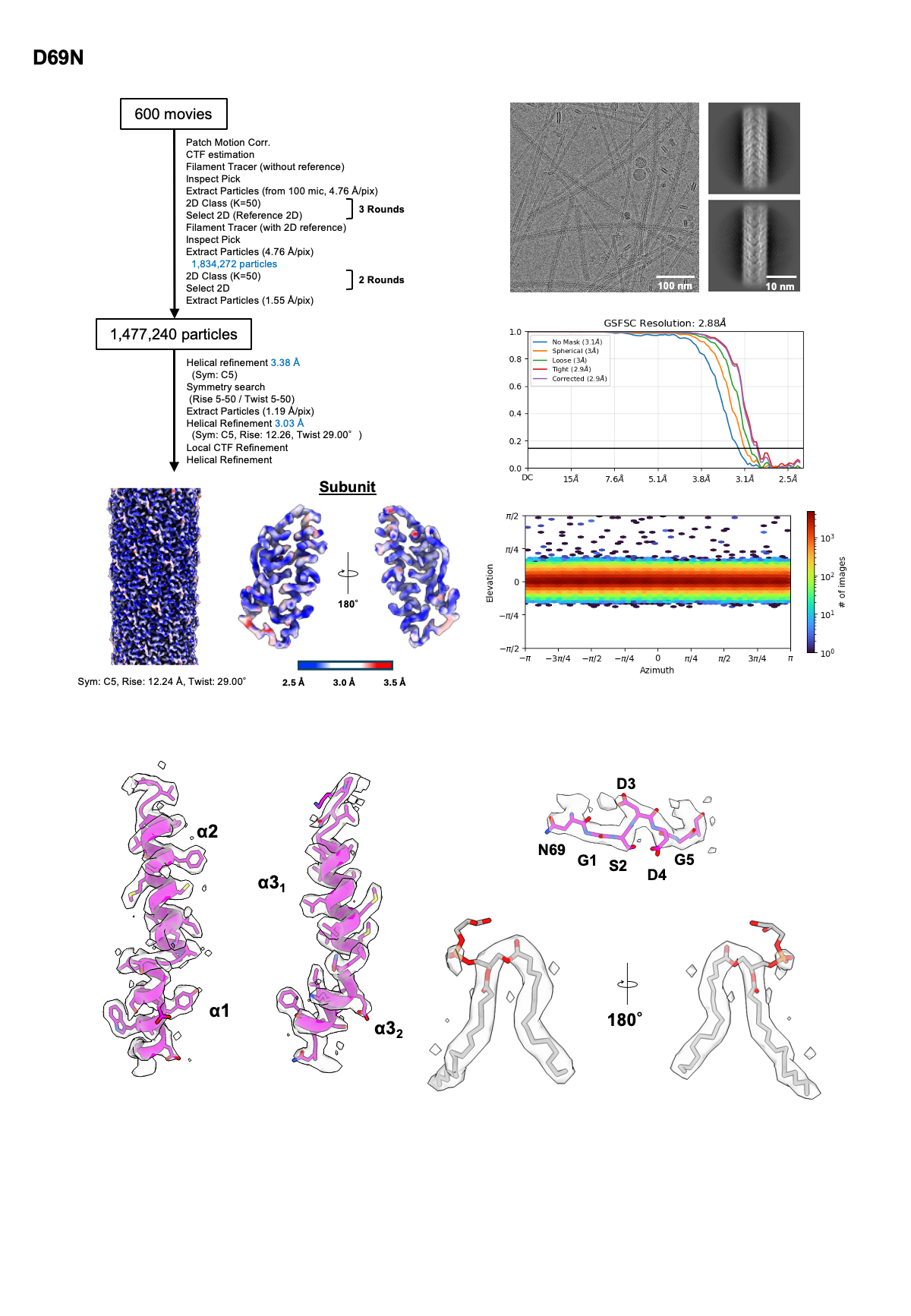


**b**
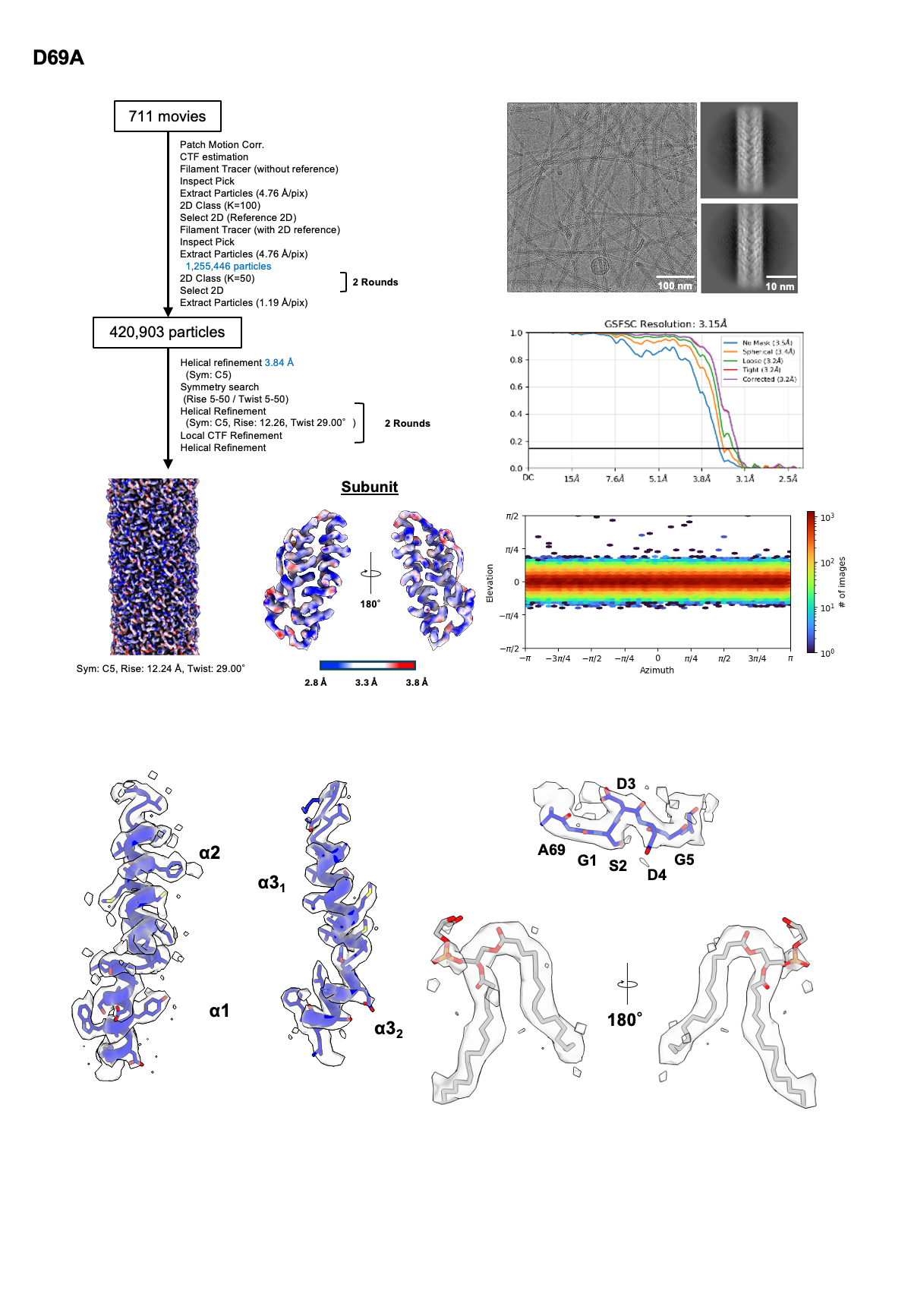


**c**
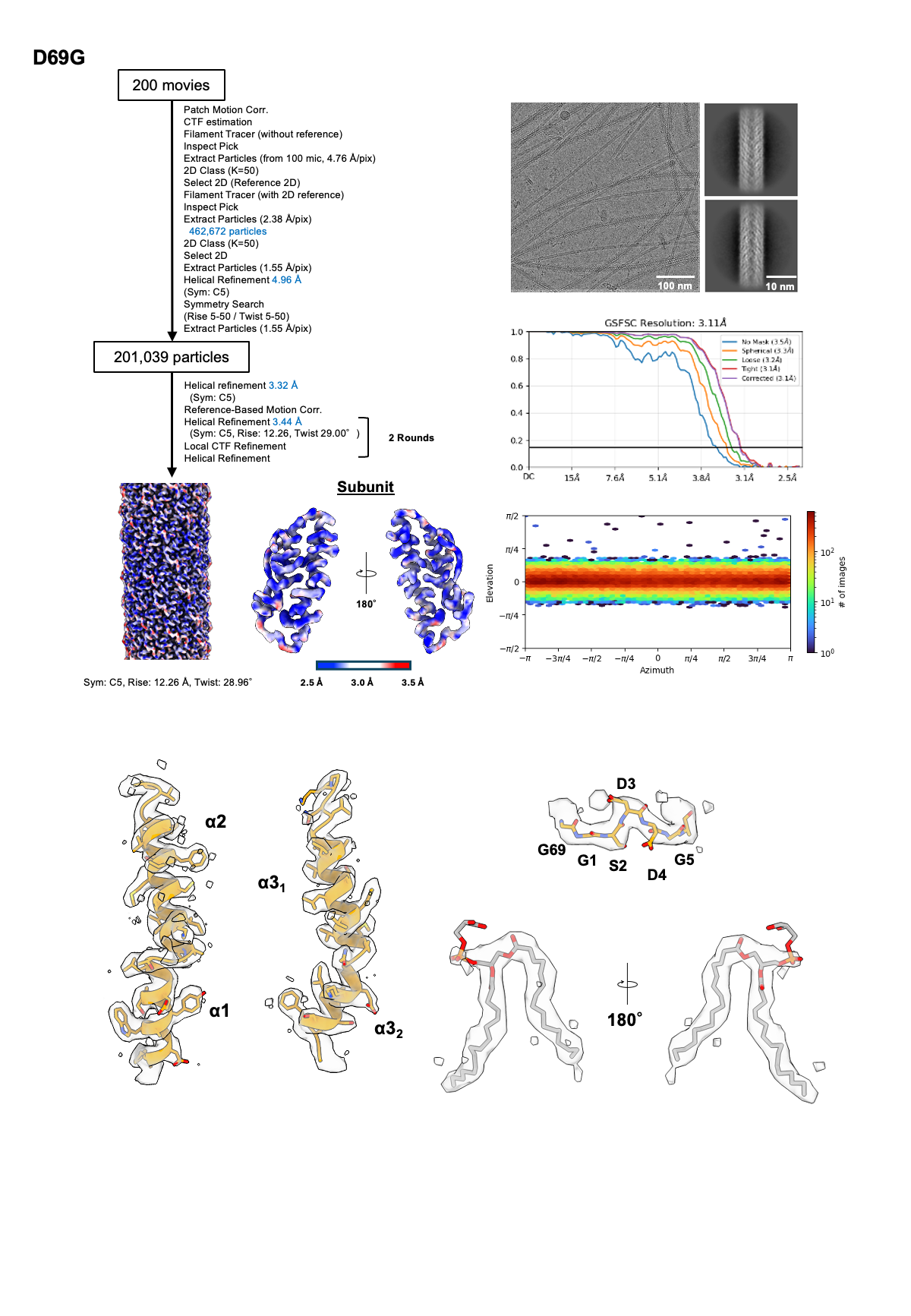


**d**
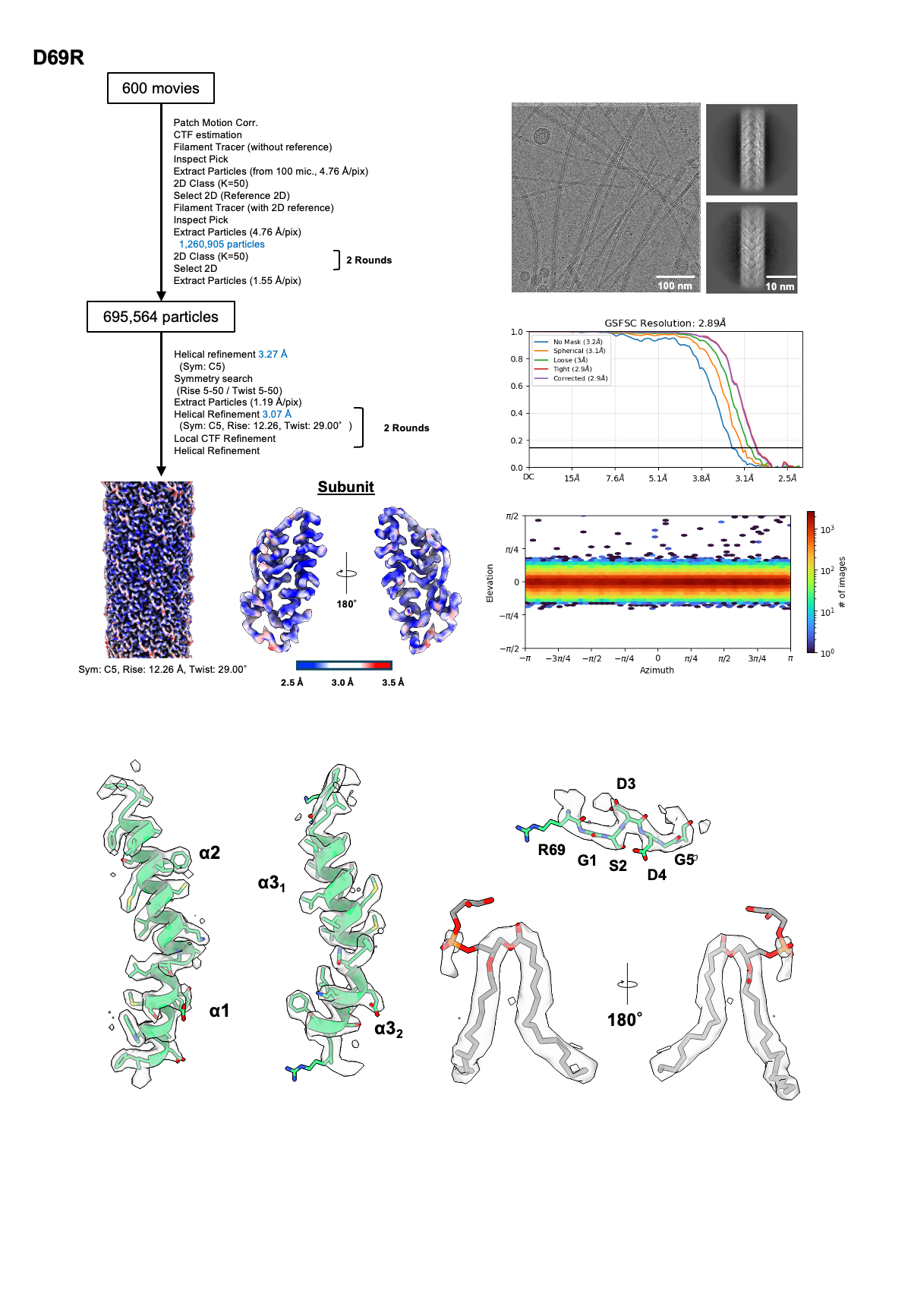


**Supplementary Figure 2**. Cryo-EM data processing workflow for the H pilus mutants; (a) D69N, (b) D69A, (c) D69G and (d) D69R. All the processing was performed in cryoSPARC (v.4.6). The gold-standard FSC curve of the final map is shown. The resolution cut-off was at FSC=0.143. The maps are coloured according to the local resolution calculated with the MonoRes option in cryoSPARC. The structure model and map are overlaid, shown at the bottom.

**
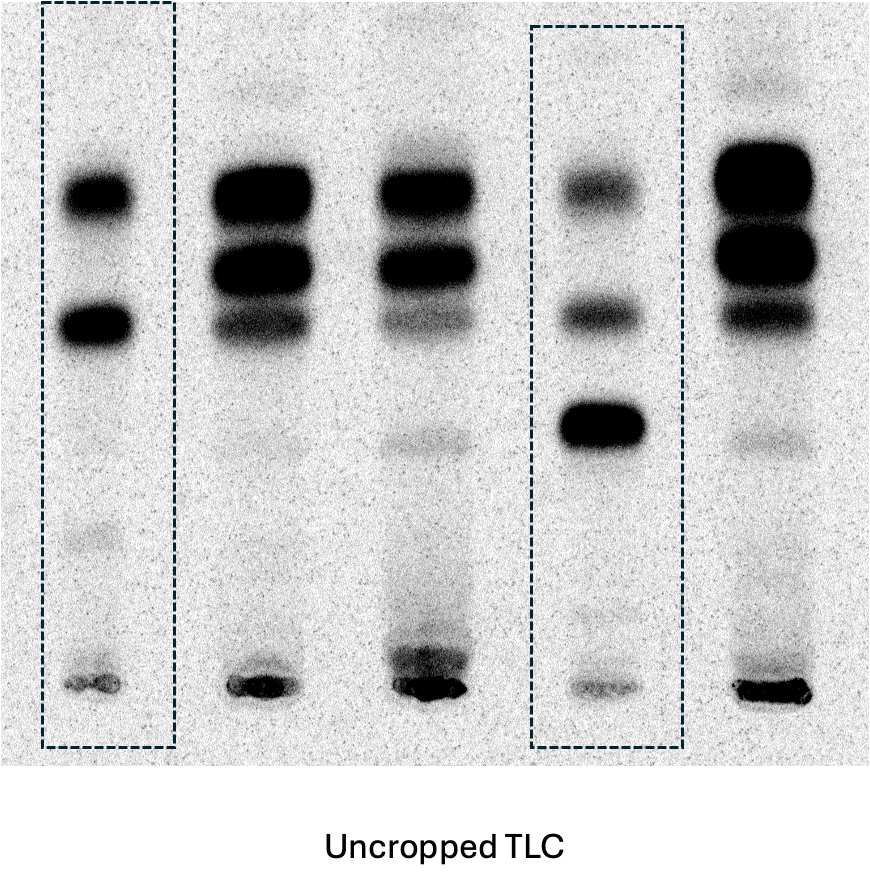
**

**Supplementary Figure 3.** Uncropped TLC related to Figure 5. Dashed boxes indicate lanes used in Figure 5.

**Supplementary Table** **1.** Primers used in this study.

| **Primers** | **Sequences** | **Description** |
| --- | --- | --- |
| F_WT_trhA | GTTATCCCTAAGTGACTTTTACGATAAGC | Amplifies the *trhA* gene from R27 plasmid |
| R_WT_trhA | CTCTATTCTTGATGGAGCTAGGGATAAC |  |
| F_D69_SDM | GCTGGTATTCCTCTGTGA | Amplifies liner vector pSEVA612s for all the D69X mutagenesis |
| R_69N_SDM | GTTCAGGAACGAGCTGA | Amplifies liner vector pSEVA612s for the D69N mutagenesis |
| R_69R_SDM | GCGCAGGAACGAGCTGA | Amplifies liner vector pSEVA612s for the D69R mutagenesis |
| R_D69A_SDM | CGCCAGGAACGAGCTG | Amplifies liner vector pSEVA612s for the D69A mutagenesis |
| R_D69G_SDM | GCCCAGGAACGAGCTGATA | Amplifies liner vector pSEVA612s for the D69G mutagenesis |
| F_D69K_SDM | CTCGTTCCTGaaaGCTGGTATTC | Amplifies liner vector pSEVA612s for the D69K mutagenesis |
| **F_G1S_SDM** | **TGCGTACGCGagcTCCGATGATG** | **Amplifies liner vector pSEVA612s for the G1S mutagenesis** |
| **R_G1S_SDM** | AAACTGCAATTTGCTAAAACCAAGAG |  |
| **F_G1K_SDM** | TGCGTACGCGaaaTCCGATGATG | **Amplifies liner vector pSEVA612s for the G1K mutagenesis** |
| **R_G1K_SDM** | AAACTGCAATTTGCTAAAAC |  |
| **F_G1D_SDM** | TGCGTACGCGgatTCCGATGATG | **Amplifies liner vector pSEVA612s for the G1D mutagenesis** |
| **R_G1D_SDM** | AAACTGCAATTTGCTAAAACCAAGAGAATAAAC |  |
| **F_IncH pilin seq** | GCTCCATCAAGAATAGAGG | Sanger sequencing primer to check the amino acid substitution |
| **F_IncH pilin seq** | CCAATGCGTTTTCGTGA |  |

**Supplementary Table** **2.** Cloning plasmids used for mutagenesis.

| **Vector** | **Description** |
| --- | --- |
| pACBSR | SmR; expresses I-SceI and lambda-red induced by L-Ara. |
| pSEVA612S | GmR; integrative plasmid (ori R6K) that harbours the oriT for tri-parental mating. |
| pSEVA612S_*trhA* WT | pSEVA612S derivative; used for the building the site-direct mutagenesis constracts for *trhA* from R27 |
| pSEVA612S_Δ *trhA* | pSEVA612S derivative; used for the deletion of *trhA* gene from R27 |
| pSEVA612S_*trhA* D69A | pSEVA612S derivative; used for amino acid substitution of D69A from TrhA |
| pSEVA612S_*trhA* D69R | pSEVA612S derivative; used for amino acid substitution of D69R from TrhA |
| pSEVA612S_*trhA* D69G | pSEVA612S derivative; used for amino acid substitution of D69G from TrhA |
| pSEVA612S_*trhA* D69N | pSEVA612S derivative; used for amino acid substitution of D69N from TrhA |
| pSEVA612S_*trhA* D69K | pSEVA612S derivative; used for amino acid substitution of D69K from TrhA |
| pSEVA612S_*trhA* G1K | pSEVA612S derivative; used for amino acid substitution of G1K from TrhA |

**Supplementary Table** **3.** Conjugative plasmids and strains used and generated in this study.

| **Plasmids** | **Description** |
| --- | --- |
| R27 | Prototype plasmid for IncH, and it is a derepressed plasmid. Its cyclic region is 67F-74L. |
| R27 Δ67-74 | R27 plasmid with a deletion of TrhA amino acid from 67-74, considers as no conjugative pilus production. |
| R27 D69A | R27 plasmid with an amino acid substitution of TrhA D69A |
| R27 D69N | R27 plasmid with an amino acid substitution of TrhA D69N |
| R27 D69G | R27 plasmid with an amino acid substitution of TrhA D69G |
| R27 D69R | R27 plasmid with an amino acid substitution of TrhA D69R |
| R27 D69K | R27 plasmid with an amino acid substitution of TrhA D69K |
| R27 G1K | R27 plasmid with an amino acid substitution of TrhA G1K |

| **Strains** | **Description** |
| --- | --- |

| CC118 λ pir | Expresses the Pi protein for the replication of plasmids with the R6K origin. |
| --- | --- |
| *E. coli* 1047 pRK2013 | Triparental conjugation helper strain. Kanamycin resistant |

| *E. coli* | *E. coli* K-12 strain |
| --- | --- |
| *E. coli -trp* | Donor strain for conjugation assays. It is *trp*- mutant, won’t be able to grow on the minimal media. |
| *K. pneumoniae* | ICC8001. Parental wild type (WT) strain of K. pneumoniae ATCC43816 serially passaged in vitro on Rifampicin(100μg/ml) followed by two passages in BALB/c mice. |
| *E. cloacae* | *Enterobacter cloacae* ATCC13047 |
| *C. amalonaticus* | CMS57 isolate 57.41.1 / Citrobacter.amalonaticusC3H ICC3000 |
| EPEC | *Enteropathogenic E. coli* e2348/69 |
| AL95 | A *E. coli* strain lacking phosphatidylethanolamine; Δ*pssA* |
| W3110 | The wild type *E. coli* strain for AL95 |

**Supplementary Table** **4.** Data collection, processing and refinement statistics for the H pilus mutants.
